# Deferiprone and Gallium-Protoporphyrin Have the Capacity to Potentiate the Activity of Antibiotics in *Staphylococcus aureus* Small Colony Variants

**DOI:** 10.3389/fcimb.2017.00280

**Published:** 2017-06-22

**Authors:** Katharina Richter, Nicky Thomas, Guimin Zhang, Clive A. Prestidge, Tom Coenye, Peter-John Wormald, Sarah Vreugde

**Affiliations:** ^1^Department of Surgery, Otolaryngology Head and Neck Surgery, Basil Hetzel Institute for Translational Health Research, The Queen Elizabeth Hospital, University of AdelaideAdelaide, SA, Australia; ^2^School of Pharmacy and Medical Sciences, University of South AustraliaAdelaide, SA, Australia; ^3^Adelaide Biofilm Test Facility, Sansom Institute for Health Research, University of South AustraliaAdelaide, SA, Australia; ^4^Department of Otolaryngology Head and Neck Surgery, Tianjin First Center HospitalTianjin, China; ^5^ARC Centre of Excellence in Convergent Bio-Nano Science and TechnologyAdelaide, SA, Australia; ^6^Laboratory of Pharmaceutical Microbiology, Ghent UniversityGhent, Belgium

**Keywords:** biofilms, small colony variants, *Staphylococcus aureus*, iron, deferiprone, gallium-protoporphyrin, wound models, *Caenorhabditis elegans*

## Abstract

Small colony variants (SCVs) of bacteria like *Staphylococcus aureus* are characterized by a reduced colony size and are linked to increased antibiotic tolerance and resistance. Their altered expression of virulence factors, slow growing properties and their ability to form biofilms make the eradication of SCVs challenging. In the context of biofilm-related infectious diseases involving *S. aureus* SCVs, a therapy targeting bacterial iron metabolism was evaluated. The combination of the iron-chelator deferiprone (Def) and the heme-analog gallium-protoporphyrin (GaPP), in solution and incorporated in a surgical wound gel, was tested for activity against planktonic and sessile SCVs. To this end, the activity of Def-GaPP was assessed against planktonic *S. aureus* SCVs, as well as against *in vitro* and *in vivo* biofilms in the colony biofilm model, an artificial wound model and a *Caenorhabditis elegans* infection model. While Def alone failed to show substantial antibacterial activity, GaPP and the combination of Def-GaPP demonstrated concentration- and strain-dependent antibacterial properties. Specifically, the Def-GaPP combination significantly reduced the bacterial load in an artificial wound model and increased the survival of *S. aureus* SCV infected *C. elegans*. When Def-GaPP were combined with gentamicin or ciprofloxacin, the triple combinations exceeded the antibiofilm activity of the individual compounds in the colony biofilm model. In targeting bacterial iron metabolism, Def-GaPP showed significant activity against planktonic and sessile SCVs. Moreover, Def-GaPP could potentiate the activity of gentamicin and ciprofloxacin. Delivered in a wound healing gel, Def-GaPP showed promise as a new topical strategy against infections with *S. aureus* SCVs.

## Introduction

Small colony variants (SCVs) are naturally occurring bacteria derived from a parent strain characterized by a small colony morphology (~10% the size of the parent strain colony), slow growth rate, altered virulence factors and increased antibiotic tolerance or resistance (Proctor et al., 2006). The switch to a phenotypic altered strain can be inheritable or transient (Johns et al., 2015). *Staphylococcus aureus* SCVs are frequently non-pigmented, non-hemolytic, and dependent on external growth factors like menadione, hemin, and thymidine; they are able to survive inside eukaryotic cells, including human macrophages (von Eiff et al., 1997b; Proctor et al., 2006; Garcia L. et al., 2012). Due to their intracellular lifestyle, *S. aureus* SCVs can escape the immune attack and are protected against antibiotics leading to persistence of disease. SCVs are associated with antibiotic-refractory and recalcitrant infections, such as chronic rhinosinusitis, respiratory tract infections in cystic fibrosis, osteomyelitis, chronic wounds, or implant infections (Proctor et al., 2006; Garcia et al., 2013; Tan et al., 2014). The recovery of SCVs in routine clinical investigations requires special nutrients and prolonged culture, making SCV isolation and identification difficult (von Eiff, 2008). Prolonged treatment regimens with a variety of antibiotics are required to treat SCV-associated infections, often combined with surgical interventions. However, clinical outcomes are frequently unsatisfying due to treatment failure and recurrence of disease (Garcia et al., 2013).

Worsening the situation, SCVs can be induced by medical therapies e.g., by exposure to antibiotics such as gentamicin, or disinfectants such as triclosan (von Eiff et al., 1997a; Bayston et al., 2007). It is furthermore known that sub-therapeutic antibiotic exposure can trigger biofilm formation of *S. aureus* SCVs and their parent strains, further complicating treatment (Costerton et al., 1995; Mitchell et al., 2010a,b). Despite the clinical significance there is little knowledge concerning *S. aureus* SCV biofilms and their susceptibility to antibiotics. Innovative treatment approaches, based on compounds with a different mode of action, such as disrupting bacterial iron metabolism (Richter et al., 2017a), may be a strategy worth approaching. *S. aureus* SCVs, like all bacteria, rely on iron for growth and survival (Andrews et al., 2003), hence, the iron metabolism could be an interesting therapeutic target.

In the present study, the antimicrobial activity of a treatment combining the iron-chelator deferiprone (Def) and the heme analog gallium-protoporphyrin (GaPP) was assessed against planktonic and biofilm-associated SCVs.

## Materials and methods

### Bacterial strains

Bacterial strains were collected from the sinonasal cavities of chronic rhinosinusitis patients. Ethics approval was obtained from The Queen Elizabeth Hospital Human Research Ethics Committee, Woodville, SA, Australia. Strains included one *S. aureus* clinical isolate (parent strain P1), which was prolonged subcultured at MIC or higher concentrations of gentamicin (≥2 μg/ml). This induced small colony variants (SCV1) that featured elevated gentamicin tolerance. Another strain was a clinically isolated *S. aureus* small colony variant (SCV2).

### Characteristics of bacterial strains

#### Catalase, coagulase, and hemolytic activity

Catalase and coagulase activity of bacterial strains were determined by suspending cells in saline in a glass tube. The catalase activity was observed by gas formation following addition of hydrogen peroxide. The coagulase activity was determined by clumping of bacterial cells after addition of plasma. Hemolytic activity was determined by streaking out bacteria on sheep blood agar (Oxoid, Thermo Fisher Scientific, Scoresby, VIC, Australia). Following incubation at 37°C for 24 h the presence of a hemolysis zone was observed.

#### Auxotrophy determination

The auxotrophy type of bacteria was determined as previously described (van de Rijn and Kessler, 1980). Briefly, a bacterial suspension adjusted to 1.0 McFarland units (~3 × 10^8^ CFU/ml) was diluted 1:100 in physiological saline. One hundred microliters were spread on chemically defined medium agar (van de Rijn and Kessler, 1980). Sterile disks were infiltrated with 10 μl of hemin, menadione, and thymidine, respectively, and placed on top of the agar. Plates were incubated for 48 h at 37°C. According to the growth zones around the disks auxotrophy was determined.

#### MIC determination

The colony suspension and broth microdilution method (Wiegand et al., 2008) were used to determine the MIC of deferiprone (Def, 3-hydroxy-1,2-dimethylpyridin-4(1*H*)-one, Sigma, Castle Hill, NSW, Australia) and gallium-protoporphyrin IX (GaPP, Frontier Scientific, Logan, UT) over 48 h. The concentrations ranged from 0.08 to 40 mM Def (i.e., 10.8–5,568 μg/ml) and 0.1–50 μg/ml GaPP. In addition, the MICs of ciprofloxacin (Cip), gentamicin (Gent), mupirocin (Mup), doxycycline (Doxy), chloramphenicol (Chlor), cephalexin (Ceph), vancomycin (Van), amoxicillin (Amoxi), and streptomycin (Strep) were determined (concentration range 0.06–32 μg/ml). All compounds were purchased from Sigma unless stated differently.

#### Bacterial growth

Bacteria were suspended in tryptone soya broth (Oxoid) and adjusted to an OD 600 of 0.01. Bacterial growth was measured in a 96-well plate over 40 h at 37°C using an EnSpire Multimode Plate Reader (Perkin Elmer, Waltham, MA). Loops were taken after 24 and 40 h to determine the colony morphology.

### Hydrogel preparation

Hydrogels were prepared by adequate mixing of dextran-aldehyde, succinyl-chitosan, and a buffer solution, as previously described (Richter et al., 2017b). Def (20 mM) and/or GaPP (100 or 500 μg/ml) were incorporated in the gel and compared to blank gel and antibiotic loaded gel including 5 μg/ml ciprofloxacin (Cip) and 100 μg/ml gentamicin (Gent). Cip and Gent were chosen due to their clinical relevance as antibiotic therapies for respiratory tract, skin, blood, bone, and soft tissue infections. Furthermore, fluoroquinolone antibiotics (such as Cip) were described as being highly effective against SCVs *in vitro* and *in vivo* (Garcia L. et al., 2012; Garcia et al., 2013).

### *In vitro* activity in the colony biofilm model

A bacterial suspension was prepared in 0.9% saline and adjusted to 1.0 McFarland units (~3 × 10^8^ CFU/ml). After diluting bacteria 1:1,000 in tryptone soya broth, 2 μl were spotted on UV-sterilized Whatman polycarbonate membrane filters (0.2 μm pore size, GE Healthcare, Little Chalfont, UK) and placed on tryptone soya agar (Merritt et al., 2005). Following biofilm formation after 24 h incubation at 37°C, the membrane filters were transferred onto AB trace agar (minimal growth agar including 0.5% glucose and 0.5% peptone). One hundred microliters of freshly prepared hydrogel was placed on biofilms and incubated for 2.5 days at 37°C. The filter membranes were then aseptically transferred to new AB trace agar plates and incubated for 2.5 days at 37°C. Finally, the filters were collected in PBS to extract bacteria by vortexing (1 min) and sonication (15 min), prior to serial dilutions and plating on tryptone soya agar for CFU counting and colony morphology determination after 3 days incubation at 37°C.

The antibiofilm effect of drug loaded gels was rated using the Bliss Independence Model (Morones-Ramirez et al., 2013; Thangamani et al., 2015). The synergy of treatment combinations (Def-GaPP100, Def-GaPP100-Cip, Def-GaPP100-Gent, Def-GaPP500) was calculated according to the following equation, with values above zero corresponding to synergistic effects.

(1) $$\begin{array}{r} \text{S}=\left(\frac{a}{MG}\right)*\left(\frac{b}{MG}\right)-\left(\frac{ab}{MG}\right) \end{array}$$

*S* = synergistic effect, *a* = Log_10_ of biofilm after exposure to compound a, *b* = Log_10_ of biofilm after exposure to compound b, *ab* = Log_10_ of biofilm after exposure to treatment combination ab, *MG* = Log_10_ of untreated biofilm (maximum growth).

### *In vitro* activity in an artificial wound model

Hyaluronic acid (1.20–1.80 MDa, Lifecore Biomedical, Chaska, MN) and collagen (Corning Incorporated, Corning, NY) were used to prepare an artificial dermis (Brackman et al., 2016), which was immersed in lyophilized bovine plasma, 19 ml Bolton broth (Oxoid), 1 ml horse blood and 10 IU of heparin. Subsequently, 10 μl of an overnight culture adjusted to 1 × 10^6^ CFU/ml was spotted on top of the dermis and incubated for 24 h at 37°C. The formed biofilms were exposed to 150 μl gel (blank gel, Def, GaPP 500, Def-GaPP500, and Cip gels) for 24 h at 37°C. Following a washing step, the dermis was immersed in 10 ml of 0.9% saline to recover the bacteria by vortexing and sonication (alternating cycles of 3 × 30 s), prior to serial dilutions and plating for CFU counting. In addition, the colony morphology was determined.

### *In vivo* activity in a *C. elegans* infection model

Synchronized nematodes, *Caenorhabditis elegans* AU37 (*glp-4*; *sek-1*), were grown to L4 stage, suspended in OGM medium (95% M9 buffer, 5% brain heart infusion broth, 10 μg/ml cholesterol) and added into 96-well plates with at least 20 worms per well (Brackman et al., 2011). Nematodes were infected with 25 μl of an overnight culture adjusted to 2 × 10^9^ CFU/ml in OGM medium and exposed to 25 μl of treatment (Def 20 mM, GaPP 500 μg/ml or a combination of both). Uninfected nematodes in OGM medium as well as infected but untreated nematodes were used as controls. The number of viable and dead nematodes was assessed every 24 h over 3 days incubation at 25°C. Subsequently, nematodes were first washed in M9 buffer containing 1 mM sodium azide, then washed in PBS prior to counting. The nematodes were mechanically disrupted by vortexing the worms in microtubes with 1.0 mm silicon carbide beads for 10 min (BioSpec Products, Bartlesville, OK). Serial dilutions of the supernatants were plated (tryptone soya agar with 7.5% NaCl) for CFU counting and colony morphology determination.

### Statistics and software

All experiments were conducted in triplicate and are presented as mean ± standard deviation (*SD*) or mean ± standard error of the mean (SEM). Results were analyzed using two-way analysis of variance with Dunnett's test (GraphPad Prism version 7.02, GraphPad Software, La Jolla, CAL). Statistical significance was assessed at the 95% confidence level.

## Results

### Characteristics of bacterial strains

**Figure 1:**
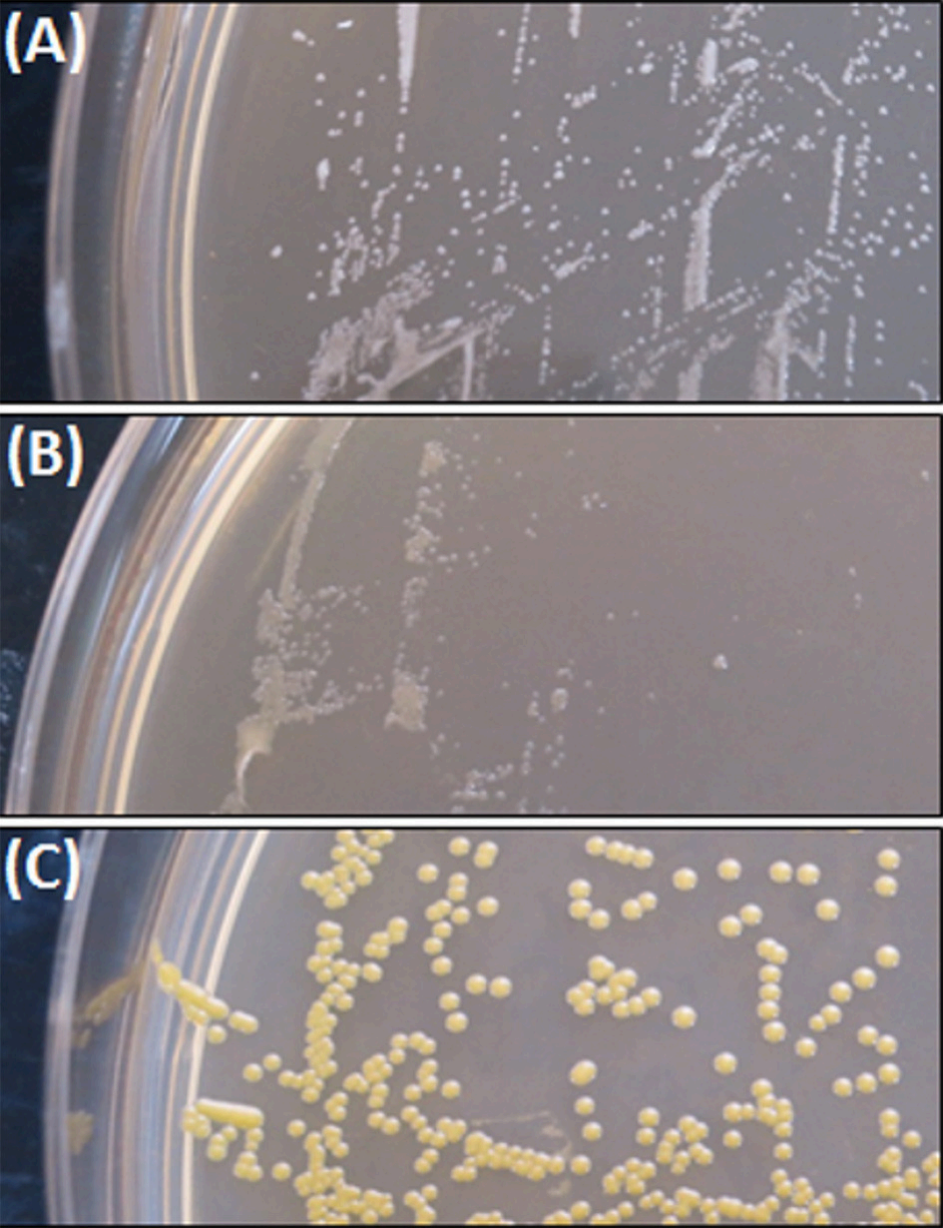
*S. aureus* small colony variant SCV1 **(A)**, SCV2 **(B)**, and parent strain P1 **(C)**.

**Figure 2:**
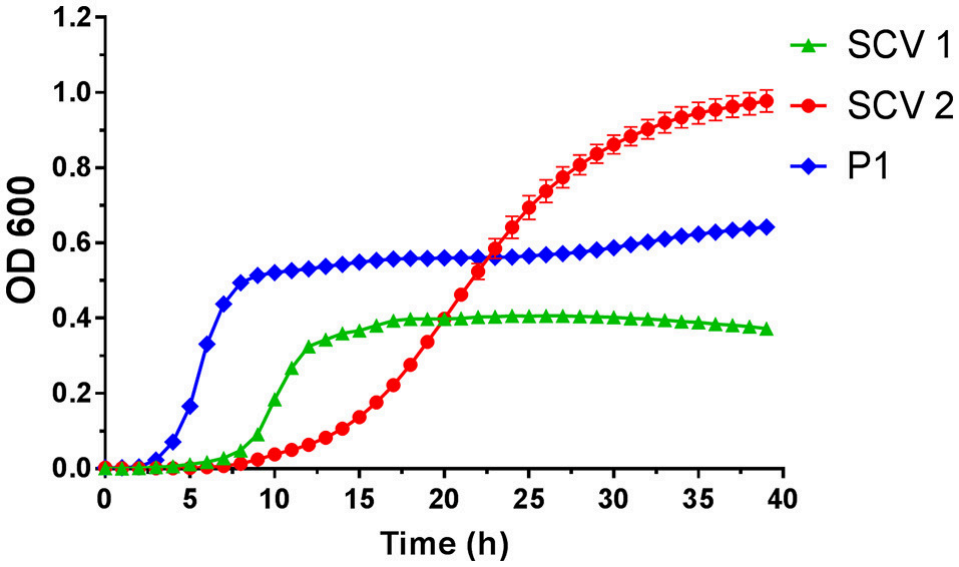
Growth curves of small colony variant SCV1 (green triangles), SCV2 (red dots) and parent strain P1 (blue diamonds).

The colony morphology of bacterial strains is shown in Figure 1 and the growth curves are displayed in Figure 2. A summary of bacterial characteristics is shown in Table 1.

**Table 1:** Characteristics of small colony variant SCV1, SCV2, and parent strain P1, including catalase, coagulase and hemolytic activity, auxotrophy type, as well as MICs (in μg/ml) of deferiprone (Def), gallium-protoporphyrin (GaPP), the combination of both compounds, ciprofloxacin (Cip), gentamicin (Gent), mupirocin (Mup), doxycycline (Doxy), chloramphenicol (Chlor), cephalexin (Ceph), vancomycin (Van), amoxicillin (Amoxi), and streptomycin (Strep).

	**SCV 1**	**SCV 2**	**P1**
Catalase	Positive	Negative	Positive
Coagulase	Positive	Positive	Negative
Hemolysis	Positive	Negative	Positive
Auxotrophy	Hemin/menadione	Thymidine	Hemin/menadione
Def	1,392	348	5,568
GaPP	6.25	>50	12.5
Def-GaPP	348/3.125	174/1.56	1,392/12.5
Cip	2	4	0.5
Gent	16	16	2
Mup	0.25	>32	1
Doxy	4	0.5	0.25
Chlor	8	4	8
Ceph	2	>32	4
Van	1	>32	2
Amoxi	32	>32	4
Strep	>32	>32	32

SCV1 was observed to be a catalase positive, coagulase positive, and hemolysis positive strain with hemin/menadione auxotrophy. SCV2 was determined as a catalase negative, coagulase positive, and hemolysis negative strain with thymidine auxotrophy. P1 was identified to be catalase positive, coagulase negative, and hemolysis positive with hemin/menadione auxotrophy. As depicted in Figure 2, during 24 h SCV1 and SCV2 showed a slower growth rate and a lower OD 600 value than the parent strain P1. While SCV1 reached stationary and decline phase after 24 h, SCV2 continued to grow reaching an OD 600 of 0.95 after 40 h. Loops taken after 24 h revealed a small colony morphology for both SCV1 and SCV2, while after 40 h SCV2 presented as a mix of small colonies and very few normal sized colonies. SCV1 showed a small morphology after 40 h.

SCV1 and SCV2 were 4- and 16-fold more susceptible to Def and 4- and 8-fold less susceptible to Cip and Gent compared to the parent strain P1. SCV1 and P1 had low MICs for GaPP (6.25 and 12.5 μg/ml, respectively), while SCV2 was observed to have a MIC above 50 μg/ml for GaPP. The MICs for the Def-GaPP combination were typically lower than the MICs for individual compounds, however, the extent of this difference was strain-dependent. As displayed in Table 1, SCV1 was susceptible to Mup and Van, and showed increasing MIC values for Ceph, Doxy, and Chlor, and was not susceptible to Amoxi and Strep. SCV2 was susceptible to Doxy, less susceptible to Chlor and not susceptible to Mup, Ceph, Van, Amoxi, and Strep. P1 showed low MIC values for Doxy and Mup, and increasing MICs for Van, Ceph, Amoxi, and Chlor, and was not susceptible to Strep.

### Colony biofilm model

**Figure 3:**
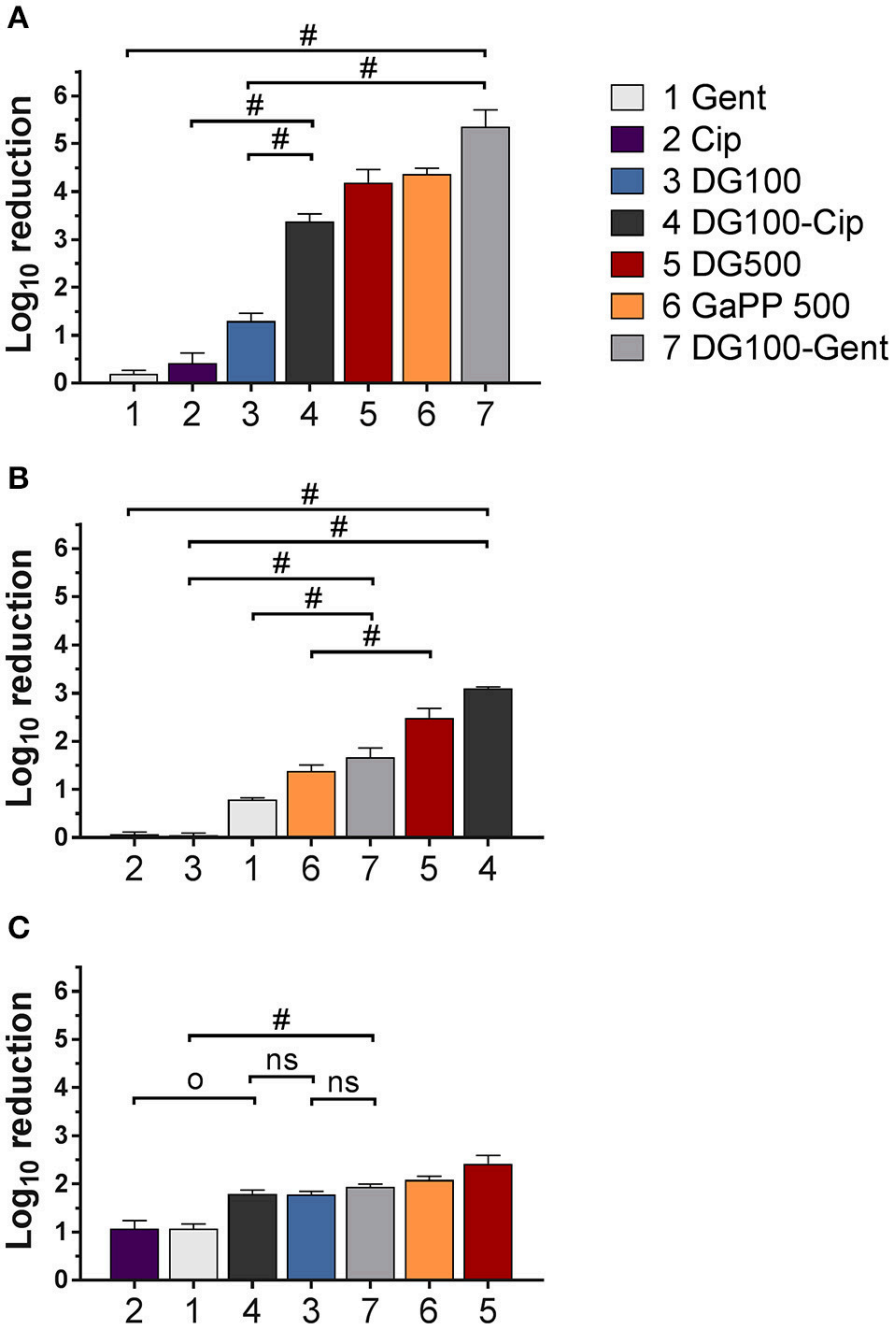
Log_10_ reduction of small colony variant SCV1 **(A)**, SCV2 **(B)** and parent strain P1 **(C)** colony biofilms after exposure to drug loaded hydrogels compared to untreated control. 1: Gentamicin (Gent) 100 μg/ml (light gray), 2: Ciprofloxacin (Cip) 5 μg/ml (purple), 3: Deferiprone (Def, 20 mM)-Gallium-protoporphyrin (GaPP) 100 μg/ml (blue), 4: Def-GaPP100-Cip (black), 5: Def-GaPP500 (red), 6: GaPP 500 (orange), 7: Def-GaPP100-Gent (dark gray). Data represent the mean ± *SD* of three biological replicates. ^*O*^*p* < 0.001; ^#^*p* < 0.0001; ns-not statistically significant.

The blank gel and the Def gel showed no antibiofilm activity (data not shown), while the GaPP gel demonstrated a concentration- and strain-dependent effect. Gel loaded with a low concentration of GaPP (100 μg/ml) showed a log_10_ reduction of 1.7 and 1.8 against biofilms of SCV1 and its parent strain P1, respectively, but no antibiofilm activity against SCV2 (data not shown), while at 500 μg/ml GaPP showed a log_10_ reduction of 4.3, 1.4, and 2.0 in SCV1, SCV2, and P1 biofilms (Figure 3). Cip and Gent loaded gels were observed to have only a minor effect against SCV1, SCV2, and P1 (log_10_ reduction of 0.4, 0.1, and 1.1, respectively for Cip and 0.2, 0.8, and 1.1, respectively for Gent). A small colony morphology of both SCV1 and SCV2 has been observed when analyzing CFUs after treatment exposure.

**Figure 4:**
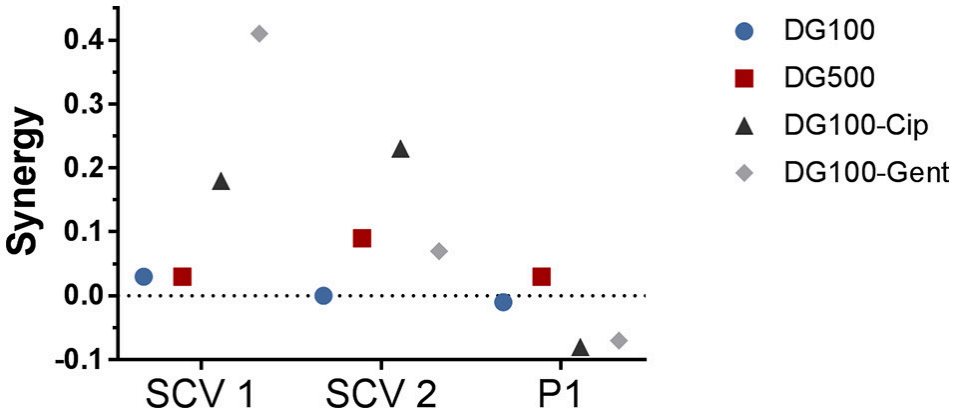
Synergy of treatment combinations against small colony variant SCV1, SCV2 and parent strain P1 colony biofilms. Deferiprone 20 mM (Def)-Gallium-protoporphyrin (GaPP) 100 μg/ml (blue circles), Def-GaPP500 μg/ml (red squares), Def-GaPP100-ciprofloxacin 5 μg/ml (black triangles), Def-GaPP100-gentamicin 100 μg/ml (gray diamonds). The higher the value the higher the degree of synergy.

The hemin auxotroph SCV1 (Figure 3A) showed increased susceptibility to gel loaded with Def-GaPP100-Cip, Def-GaPP100-Gent, GaPP 500, and Def-GaPP500 (log_10_ reduction of 3.4, 5.4, 4.3, and 4.4, respectively), and decreased susceptibility to monotherapy with Cip or Gent compared to its parent strain P1. Interestingly, Def-GaPP100 combined with Gent showed a high degree of synergy (Figure 4) and significant activity against the highly Gent-tolerant SCV1 compared to Gent alone and Def-GaPP100 (log_10_ reduction of 5.4 for Def-GaPP100-Gent vs. 0.2 for Gent and 1.3 for Def-GaPP100, *p* < 0.0001).

The thymidine auxotroph SCV2 (Figure 3B) showed elevated susceptibility to gel incorporating Def-GaPP100-Cip, Def-GaPP100-Gent, GaPP 500, and Def-GaPP500 (log_10_ reduction of 3.1, 1.7, 1.4, and 2.5, respectively), and no susceptibility to Cip or Def-GaPP100. Notably, Def-GaPP100 combined with Cip showed high synergy (Figure 4) and significant activity against SCV2, manifestly exceeding the effect of the individual compounds and Def-GaPP100 (log_10_ reduction of 3.1 for Def-GaPP100-Cip vs. 0.1 for Cip and 0.1 for Def-GaPP100, *p* < 0.0001).

Against the hemin auxotroph P1 (Figure 3C) the combination of Def-GaPP100 gel with Cip or Gent showed significantly higher activity than the antibiotics alone (log_10_ reduction of 1.8 for Def-GaPP100-Cip vs. 1.1 for Cip, *p* < 0.001; and 1.9 for Def-GaPP100-Gent vs. 1.1 for Gent, *p* < 0.0001). However, no synergistic effect was observed compared to the Def-GaPP100 gel (1.8 log_10_ reduction). The highest activity against P1 was achieved with Def-GaPP500 gel (2.4 log_10_ reduction).

### Macroscopic biofilm analysis

**Figure 5:**
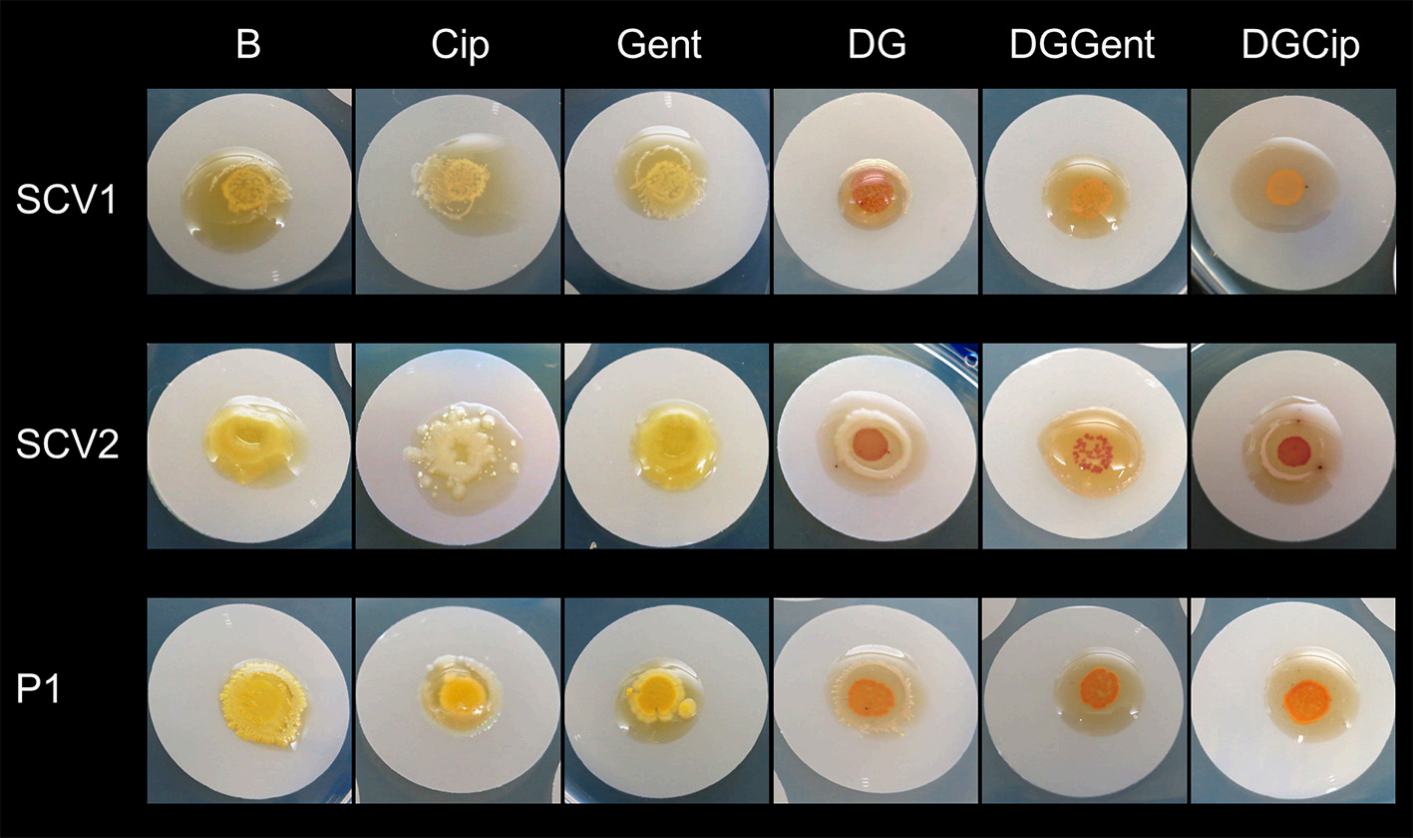
Inhibitory effect of drug loaded hydrogels on biofilms after 5 days exposure. Elevated biofilm inhibition was observed for gels containing deferiprone, gallium-protoporphyrin and ciprofloxacin or gentamicin (DGCip, DGGent). Strains used: Small colony variant SCV1, SCV2 and parent strain P1. Hydrogels- B, Blank control gel; Cip, Ciprofloxacin 5 μg/ml; Gent, Gentamicin 100 μg/ml; DG, Deferiprone 20 mM-Gallium-protoporphyrin 100 μg/ml; DGGent, Def-GaPP100-Gent; DGCip, Def-GaPP100-Cip.

The antibiofilm activity of loaded hydrogels was macroscopically analyzed over 5 days of treatment exposure. All biofilms grew extensively when exposed to blank gel and gel incorporating Def (not shown), Cip and Gent (Figure 5). A concentration- and strain-dependent antibiofilm effect was apparent after exposure to gels incorporating GaPP (not shown), Def-GaPP, Def-GaPP-Cip, and Def-GaPP-Gent. While Def-GaPP100 gel moderately inhibited bacterial growth, the combination of Def-GaPP100 with either Cip or Gent resulted in a substantial antibiofilm effect (Figure 5).

### Artificial wound model

**Figure 6:**
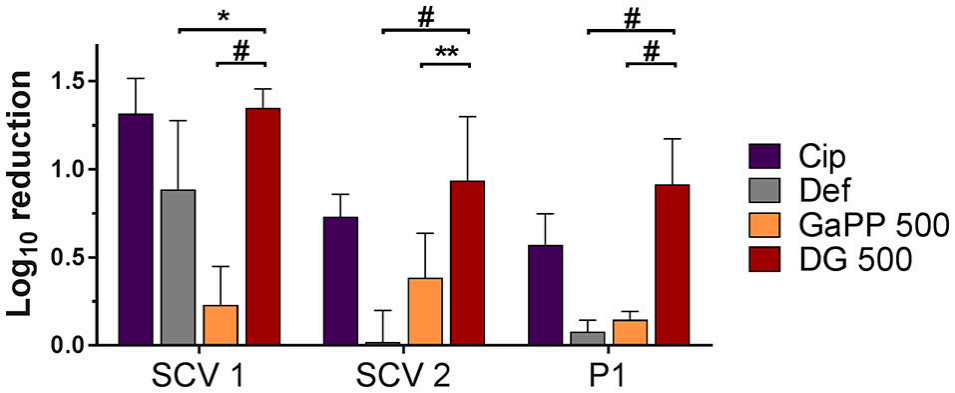
Effects of hydrogels in an artificial wound model compared to untreated control. Log_10_ reduction of small colony variant SCV1, SCV2, and parent strain P1 after exposure to hydrogels loaded with ciprofloxacin 5 μg/ml (purple), deferiprone 20 mM (Def, gray), gallium-protoporphyrin 500 μg/ml (GaPP, orange), and Def-GaPP500 (red). Data represent the mean ± *SD* of three biological replicates. ^*^p < 0.05; ^**^p < 0.01; ^#^*p* < 0.0001.

Biofilms grown on an artificial dermis were exposed to drug loaded hydrogels to determine the antibiofilm activity in an *in vitro* wound model (Figure 6). The visual analysis of CFUs after treatment exposure confirmed a small colony morphology for the majority of both SCV1 and SCV2. The untreated control and the blank gel showed similar growth of all biofilms, indicating no antibiofilm effect of the blank gel. The Def gel demonstrated substantial antibiofilm activity against SCV1 (log_10_ reduction of 0.9), but failed to be effective against P1 and SCV2. The GaPP 500 gel showed minor antibiofilm activity with a 0.2–0.4 log_10_ reduction. In contrast, Def-GaPP500 gel showed significant antibiofilm effects against SCV1, SCV2, and P1 with a 1.4, 1.0, and 0.9 log_10_ reduction, respectively, thereby demonstrating significantly higher activity than the individual compounds (*p* < 0.05–0.0001) and slightly higher activity than Cip gel (Figure 6).

### *In vivo* infection model in *C. elegans*

**Figure 7:**
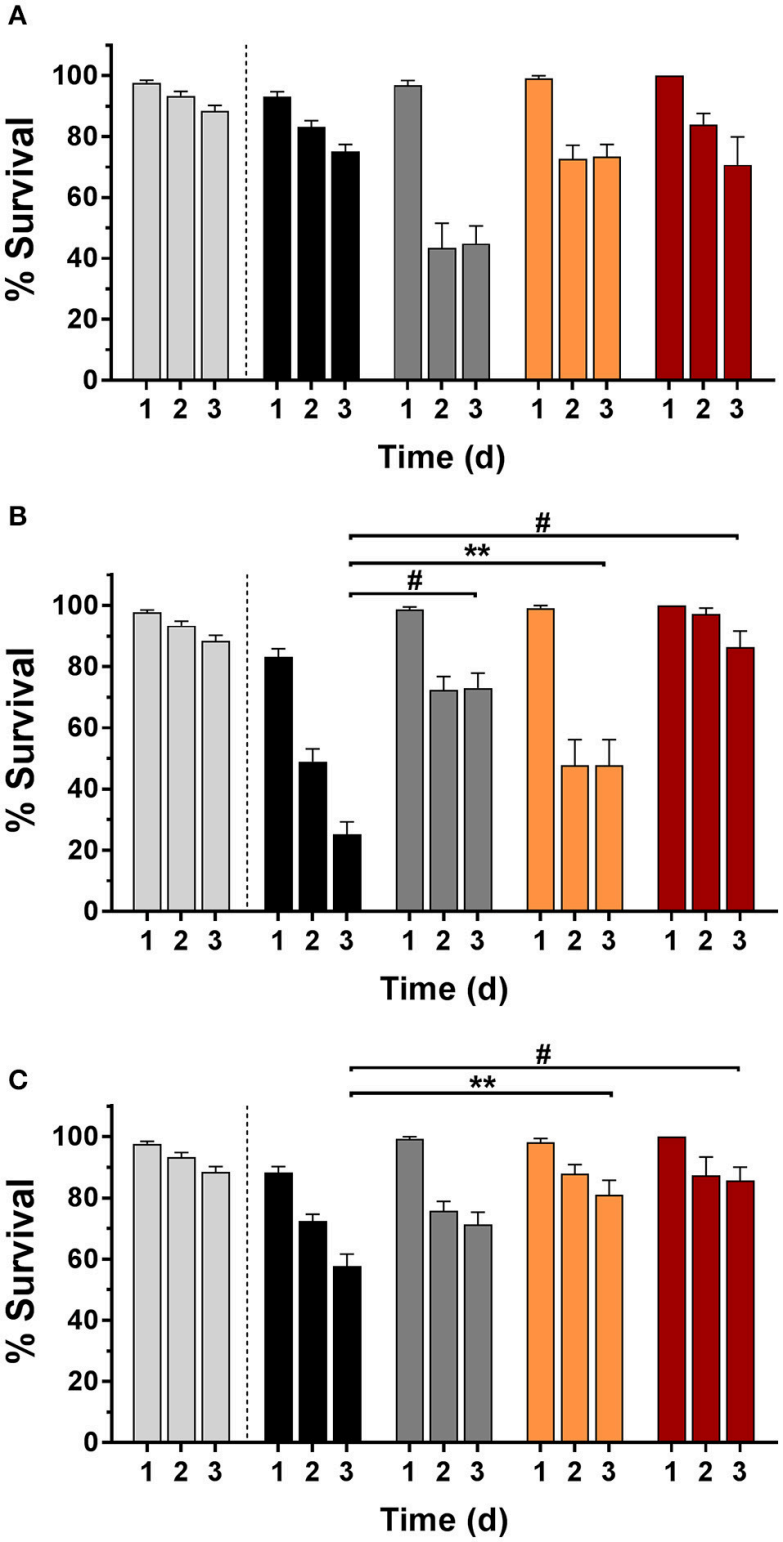
*C. elegans* survival (%) over 3 days in uninfected controls (light gray) and after infection (black bars) with small colony variant SCV1 **(A)**, SCV2 **(B)** or parent strain P1 **(C)** and treatment with loaded hydrogels: deferiprone 20 mM (Def, dark gray), gallium-protoporphyrin 500 μg/ml (GaPP, orange), and Def-GaPP500 (red). Data represent the mean ± SEM of at least six biological replicates. ^**^p < 0.01; ^#^*p* < 0.0001.

Nematodes were infected with bacteria and their survival rate was determined with and without Def, GaPP 500, or Def-GaPP500 treatment (Figure 7). The worm killing was strain-dependent with 75, 25, and 57% survival in SCV1, SCV2, and P1 infected worms, respectively, while uninfected controls showed 88% survival over 3 days. When worms were exposed to Def, 45, 73, and 71% of SCV1, SCV2, and P1-infected nematodes survived, while 73, 48, and 81% infected worms survived when treated with GaPP 500. The combination of Def-GaPP500 showed a similar survival rate in SCV1 and P1 infected worms as GaPP 500 alone (71 and 87% survival, respectively). In contrast, the survival rate of SCV2 infected worms was substantially higher (86% survival) when treated with Def-GaPP500 compared to the individual treatments. Compared to the uninfected control the Def-GaPP500 treatment achieved similar survival rates in SCV2 and P1 infected worms. Furthermore, the survival of SCV1 and P1 infected worms after GaPP 500 treatment was not different to uninfected controls (Figure 7).

**Figure 8:**
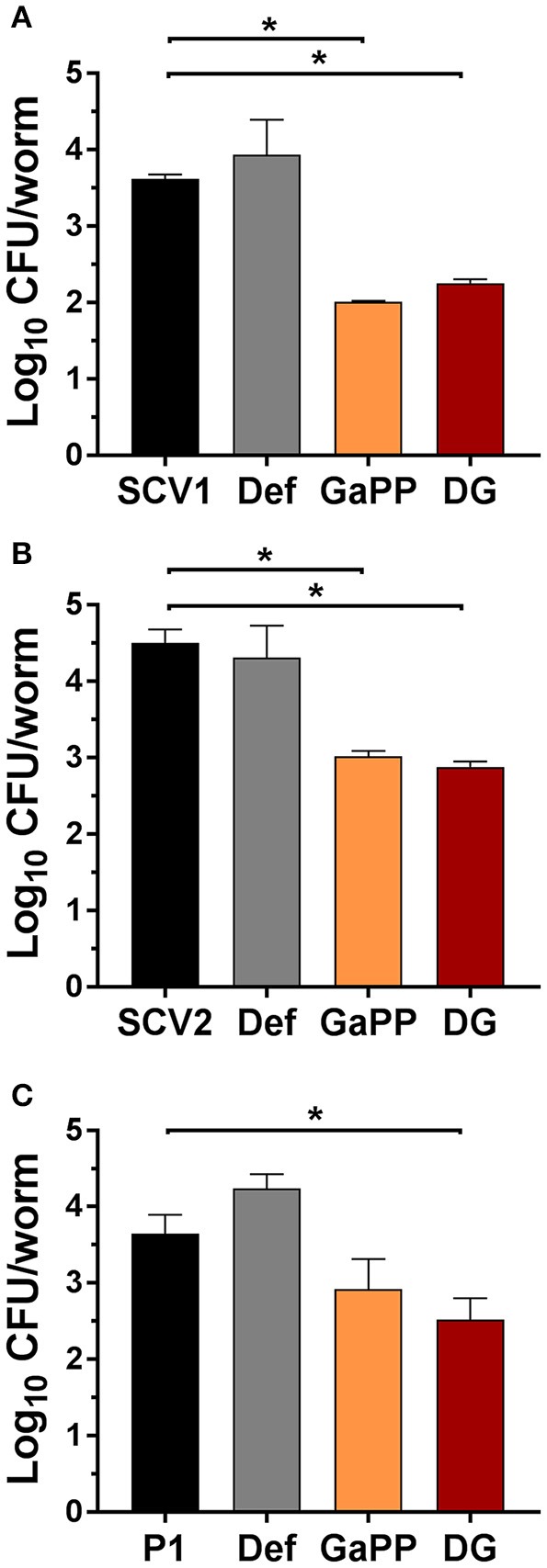
Log_10_ of CFU per *C. elegans* worm after 3 days infection (black bars) with small colony variant SCV1 **(A)**, SCV2 **(B)** or parent strain P1 **(C)** and treatment with drug loaded hydrogels- Def: deferiprone 20 mM (gray), GaPP, gallium-protoporphyrin 500 μg/ml (orange) and DG, Def-GaPP500 (red). Data represent the mean ± *SD* of at least 6 biological replicates. ^*^p < 0.05.

Following 3 days of infection the bacterial load per worm was quantified (Figure 8) by enumeration of CFU. Small colony phenotypes of SCV1 and SCV2 were observed. Consistent with results in the colony biofilm model and wound model, the treatment with Def alone showed no significant effect and failed to reduce the CFU per worm. In contrast, both GaPP 500 and Def-GaPP500 showed a significant (*p* < 0.05) reduction of the bacterial load, resulting in a log_10_ of 2.0 for GaPP and 2.3 for Def-GaPP in SCV1 infected worms (SCV1 infection control: log_10_ of 3.6 CFU/worm), 2.9 and 2.5 in P1 infected worms (P1 infection control: log_10_ of 3.6 CFU/worm), and 3.0 and 2.8 in SCV2 infected worms (SCV2 infection control: log_10_ of 4.5 CFU/worm).

## Discussion

*S. aureus* SCVs in planktonic and biofilm form have a global significance in the clinical environment being associated with treatment failure and recurrence of disease (Proctor et al., 2006; Garcia et al., 2013). Treatments are mostly based on antibiotics, however, the low growth rate, reduced antibiotic susceptibility and emerging resistance of SCVs pose a challenge for efficient medical therapies. In the present study, an alternative treatment that relies on the disruption of bacterial iron metabolism was evaluated. As previously described, the iron chelator Def and the heme analog GaPP show synergistic effects against *S. aureus* biofilms *in vitro* (Richter et al., 2016). Herein, the activity of Def-GaPP against planktonic and biofilm-associated SCVs and a parent strain with elevated antibiotic tolerance/resistance was evaluated *in vitro* and *in vivo*.

The Def-GaPP treatment exhibits antimicrobial activity based on the initial iron-chelation by Def, followed by iron depletion-induced upregulation of the bacteria's iron acquisition systems (Haley and Skaar, 2012). The latter are exploited by the heme analog GaPP mimicking heme as the preferred iron source of *S. aureus* (Stojiljkovic et al., 1999; Skaar et al., 2004; Weinberg, 2009). Inside bacteria, GaPP disrupts the iron metabolism vital for bacterial growth, survival, and virulence (Weinberg, 2009; Skaar, 2010). Unlike heme, GaPP lacks the ability to transfer electrons, hence, GaPP cannot be part of redox reactions required for respiration, ATP production and DNA synthesis. Furthermore, GaPP cannot be cleaved by bacterial enzymes and cannot be utilized as nutrient source leading to starvation. In addition, GaPP is able to block efflux pumps essential for heme homeostasis resulting in an intracellular accumulation of redox-active molecules (Stojiljkovic et al., 2001; Reniere et al., 2007). The subsequent antibacterial effects are based on starvation, limited respiration and elevated production of reactive oxygen species contributing to DNA and protein damage and ultimately cell death.

As shown in the present study, the combination of Def and GaPP exhibited significant antibacterial and antibiofilm activity against clinical isolates of *S. aureus* SCVs and a parent strain. In the colony biofilm model, where compounds were delivered in a surgical wound gel, a dose-dependent antibiofilm effect of GaPP and Def-GaPP was observed (Figures 3, 5). A colony biofilm comprises of a heterogeneous consortium of sessile cells with a mixture of metabolic active bacteria on the biofilm surface and metabolic retarded/inactive bacteria on the inside. The metabolic activity arises due to different oxygen levels (aerobic conditions on the biofilm surface, micro-aerobic/anaerobic inside the biofilm) and different nutrient availability according to the location bacteria occupy. While sessile bacteria on the biofilm surface can relatively easily be targeted, bacteria deeper in the biofilm are less exposed to compounds and show reduced susceptibility (Merritt et al., 2005). The blank gel and Def gel are thought to interfere only with the biofilm surface by attaching to bacterial cell wall proteins and depriving nutrients, respectively, resulting in minor antibiofilm activity. Likewise, gels incorporating low concentrations (100 μg/ml) of GaPP showed limited activity, indicating an insufficient GaPP penetration into the colony biofilm and/or a suboptimal GaPP concentration. A higher GaPP concentration in gels (GaPP 500, Def-GaPP500) resulted in higher antibiofilm activity. It was observed that the presence of Def was crucial to facilitate a significant activity of GaPP against SCV2 biofilms, which is likely based on a Def-induced upregulation of heme acquisition systems elevating GaPP uptake into bacteria for an improved antibiofilm effect. Another reason for differences in susceptibility of the strains used in this study can rely on auxotrophy (Garcia et al., 2013). SCVs can be classified in two types of auxotrophs, namely hemin/menadione auxotrophs and thymidine auxotrophs. This in return affects the susceptibility toward antibiotics and other antibacterial compounds (Garcia et al., 2013). SCV2 was observed to be auxotrophic for thymidine, hence, less dependent on hemin/iron which can explain the poor GaPP susceptibility. SCV1 was shown to be hemin/menadione auxotrophic, thus, more susceptible to the iron depriving and heme mimicking Def-GaPP treatment.

SCVs are electron-transport-defective strains with a reduced transmembrane potential, which impedes the penetration and activity of membrane active compounds and some antibiotic classes, such as aminoglycosides and antifolate agents (von Eiff et al., 1997a; McNamara and Proctor, 2000; Baumert et al., 2002). Therefore, Gent was expected to show low activity against both planktonic SCVs as confirmed in Table 1, and sessile SCVs as seen in Figures 3, 5. Surprisingly, when Gent was combined with Def-GaPP, the triple combination showed significant antibiofilm activity (Figures 3, 5), indicating a potentiation of Gent by Def-GaPP even against the Gent-tolerant SCV1 strain. This result implies that by disrupting bacterial iron metabolism Def-GaPP made the bacteria vulnerable and increased their susceptibility to Gent for a synergistic antibiofilm effect as confirmed in Figure 4. Similar results were observed for the treatment with Cip. In line with the literature, planktonic SCVs showed higher MIC values for Cip than the parent strain (Idelevich et al., 2011) and biofilm-associated SCVs were also poorly susceptible to Cip. In contrast, the combination of Def-GaPP-Cip showed synergistic effects (Figure 4) resulting in significantly higher antibiofilm activity, even when treatment with the individual compounds was unsuccessful (Figure 3, SCV2). This confirms that the combination of drugs with different modes of action can be effective in treating bacteria with poor antibiotic susceptibility. The higher efficacy of the triple combinations Def-GaPP-Cip and Def-GaPP-Gent might rely on the synergy between reactive oxygen species (resulting from the Def-GaPP treatment) and antibiotics, as described elsewhere (Garcia L. G. et al., 2012; Garcia et al., 2013).

The antibiofilm activity of Def-GaPP was furthermore determined in an *in vitro* wound model where biofilms were grown on an artificial dermis and exposed to loaded gels. In line with results obtained in the colony biofilm model, Def-GaPP gel showed substantial antibiofilm effects, though to a lower extent. This may be the result of a nutrient-rich environment with blood/heme as favorable iron source. Bacteria recognize the tetrapyrrole ring of heme and GaPP, but can distinguish between both compounds (Stojiljkovic et al., 1999; Moriwaki et al., 2011). Therefore, GaPP gel showed only minor antibiofilm activity, in contrast to the Def-GaPP combination. The Def-induced chelation may have deprived bacteria of nutrients and increased the uptake of GaPP as heme-mimicking agent for a substantial antibiofilm effect of this “Trojan Horse” compound.

In an *in vivo* infection model in the nematode *C. elegans* the antibacterial effect of Def-GaPP was assessed. It was observed that the survival rate of infected nematodes were strain-dependent (Figure 7). Literature described that hemin/menadione auxotrophic strains were less virulent than parent strains and thymidine auxotrophic strains (Sifri et al., 2006). In line with this the order of virulence in the present study was observed to be SCV1 (hemin/menadione auxotroph) being the least and SCV2 (thymidine auxotroph) being the most virulent strain (Figure 7). The auxotrophy type can influence the strain's susceptibility to antibacterial compounds. In the colony biofilm model SCV1 was more susceptible to GaPP and Def-GaPP than SCV2, which required the combination of Def-GaPP for a substantial antibiofilm effect. Consistent with these observations similar results were observed *in vivo* in the *C. elegans* model. While infected nematodes died over time due to bacterial colonization, biofilm formation and toxin production (Sifri et al., 2005), the exposure to Def-GaPP prolonged the lifespan of all infected nematodes (Figure 7) and reduced the bacterial burden per worm (Figure 8). Treatment with GaPP alone only increased the survival rate of SCV1 and P1 infected worms, but failed in SCV2 infected worms. Therefore, combining GaPP with Def appeared to be crucial for an elevated antibacterial activity against *S. aureus* SCVs of any auxotrophy type.

## Conclusion

The present study confirmed strong antibacterial and antibiofilm properties of Def-GaPP against *S. aureus* SCVs *in vitro* and *in vivo*. When applied in a surgical hydrogel, Def-GaPP has potential to complement the gel's wound healing properties with antibacterial and antibiofilm effects and could serve as an alternative treatment for biofilm and SCV-related infections. Due to the risk of emerging antibiotic resistance, which is in particular associated with prolonged antibiotic treatment, the combination of drugs with different modes of action is advantageous. The combination of Def-GaPP with antibiotics may facilitate a multi-pronged approach to increase the treatment efficacy against otherwise antibiotic tolerant/resistant *S. aureus* SCVs. While the results of the current pilot study are encouraging, the broader applicability of Def-GaPP for the treatment of SCVs derived from other species is on the way to be validated.

## Author contributions

Conception and design of study: KR, NT, TC, and SV. Acquisition and analysis of data: KR, NT, GZ, and TC. Drafting of article and/or critical revision: KR, NT, TC, CP, PW, and SV. All authors approved the final article.

### Conflict of interest statement

Competing financial interests: PW holds a patent on the chitosan-dextran hydrogel. PW and SV hold a patent application on the treatment combination of deferiprone and gallium-protoporphyrin. The other authors declare that the research was conducted in the absence of any commercial or financial relationships that could be construed as a potential conflict of interest.
